# Spontaneous Transethmoidal Meningoceles in Adults: Case Series with Emphasis on Surgical Management

**DOI:** 10.1155/2016/3238297

**Published:** 2016-02-16

**Authors:** G. Ziade, A. L. Hamdan, M. T. Homsi, I. Kazan, U. Hadi

**Affiliations:** Department of Otolaryngology-Head and Neck Surgery, American University of Beirut Medical Center, Phase I, 6th Floor, Room C-638, Bliss Street, P.O. Box 11-0236, Beirut, Lebanon

## Abstract

*Background.* Spontaneous onset transethmoidal meningocele is a rare entity among the adult population.* Methods.* A retrospective chart review was performed and cases of adults diagnosed with spontaneous transethmoidal meningoceles from November 2000 till February 2014 were reported. Data collected included demographics, clinical presentation, diagnostic modalities, and results. Intraoperative findings, the type of surgical reconstruction performed, and the percentage of recurrence, if present, were also reported.* Results.* Ten cases of spontaneous transethmoidal meningoceles in adults were diagnosed. Eight were females and two males with a mean age of 47.5 years. All patients presented with CSF leakage with or without meningitis. They underwent a reconstruction of the base of skull defect using the temporalis fascia graft in addition to fibrin glue (Tissucol) and Surgicel (Ethicon). In two cases with a larger defect, a piece of septal bone and turbinate mucosa were applied achieving a watertight seal in all cases.* Conclusion.* Spontaneous transethmoidal meningocele in adults is a rare condition. It usually presents with clear rhinorrhea with or without meningitis and an endoscopic multilayer reconstruction is advocated for treatment of such conditions.

## 1. Introduction

A cephalocele is a herniation of cranial content through a defect in the skull base. It can consist of meninges alone and would be referred to as meningocele or it can also include brain tissue and in that case it would be described as a meningoencephalocele [1]. These herniations can occur at different locations at the level of the calvaria or the skull base, and many classifications were proposed for the skull base cephaloceles [2–6]. Sincipital cephaloceles are the anterior herniations that always present with an external mass whereas basal cephaloceles, which include the transethmoidal subcategory, are located at the level of the anterior and middle skull base and do not present with an external mass [4, 6].

Many hypotheses have been postulated on the origin of the defect; it is believed that in childhood the most common etiology is a congenital defect, while in adulthood it is mainly due to traumatic or iatrogenic causes. Therefore, spontaneous herniations in adults rarely occur [1, 5]. Different presentations have been described in the literature for spontaneous onset transethmoidal cephaloceles in the adult population such as CSF leaks, meningitis, seizures, and nasal mass [7–16]. Many endoscopic transnasal techniques for reconstruction of the skull base defect were described and all are presented as safe techniques [12, 17–28].

Spontaneous onset transethmoidal meningocele in adults is a rare condition. Kubo et al. published the largest case series on 19 cases where 17 of them had a transcranial repair of the defect [8]. The aim of this study is to list a series of spontaneous transethmoidal meningoceles among the adult population, describe their presentation and assessment, and evaluate the transnasal endoscopic management.

## 2. Methods

This study was approved by the Institutional Review Board of the American University of Beirut. This is a retrospective review of all adult patients at the American University of Beirut Medical Center and its affiliated hospitals from November 2000 through February 2014 who were diagnosed with spontaneous transethmoidal meningoencephalocele or meningocele.

Patients with history of head trauma, head and neck surgery, hydrocephalus, or destructive or erosive lesions of the skull base were excluded. Data collected included demographics, presentation, diagnostic modalities and results, intraoperative findings, the type of surgical reconstruction performed, and recurrence.

### 2.1. Surgical Technique

**Figure 1:**
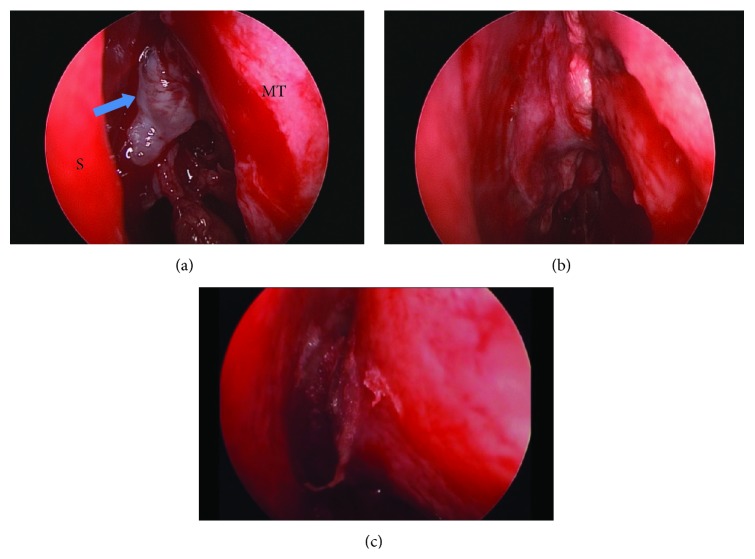
(a) Transethmoidal meningoencephalocele of the left cribriform plate (blue arrow) between the nasal septum (S) and the middle turbinate (MT). (b) Temporalis fascia applied in an overlay technique. (c) Fibrin glue applied.

The procedure began with inspection of the ethmoid roof using the zero-degree endoscope. Transethmoidal cephaloceles protrude through defects in the cribriform plate and therefore were located intranasally in the area bound by the vertical ground lamella of the middle turbinate laterally and the perpendicular plate of the ethmoid bone medially. The cephalocele was identified as a pinkish to white, translucent, fluid filled and pulsating mass (Figure 1). Occult or obvious CSF leakage was present and in some cases, topical fluorescein dye was applied confirming the site of leakage. Bipolar electrocautery was used to cauterize and fulgurate the small blood vessels and the cephalocele protruding from the defect in order to ensure a good hemostasis and avoid their retraction intracranially. A cuff of mucosa was removed from around the defect exposing the bone in order to have a good adherence between the grafted material and base of the skull. In our series, the resulting skull base defects were mainly small in size and with low flow CSF leak. Therefore, the mainstay of our reconstruction was the temporalis fascia graft, which was used as an overlay graft. Septal bone grafts, harvested from the perpendicular plate of the ethmoid bone, were used for larger defects. In two cases, an additional layer of turbinate mucosa was harvested from the inferior turbinate, reshaped, and added to the reconstructed material. In all cases, after achieving a watertight seal, fibrin glue (Tissucol) was applied in order to strengthen the reconstructed material. At the end of the procedure, a Merocel pack (Medtronic) was used as a scaffold for the reconstructed area after inserting a layer of Surgicel (Ethicon) between the pack and grafting material preventing possible adherence.

## 3. Results

Ten cases of adults diagnosed with a spontaneous onset transethmoidal cephalocele were reported. The group included 8 females (80% of patients) and 2 males (20% of patients). The median age was 47.5 years and the age range was between 27 and 68 years (the mean age was 49 ± 12.5 years). Patients presented with CSF leakage with or without meningitis. The diagnosis of CSF leakage was confirmed by the presence of beta-2-transferrin in the collected fluids. Computed Tomography (CT) and Magnetic Resonance (MR) imaging were used as radiological diagnostic modalities identifying the site of leakage. In view of the retrospective type of the study, CT myelography was also used in few cases in order to localize the site of defect. All patients were treated using a transnasal endoscopic approach.

**Figure 2:**
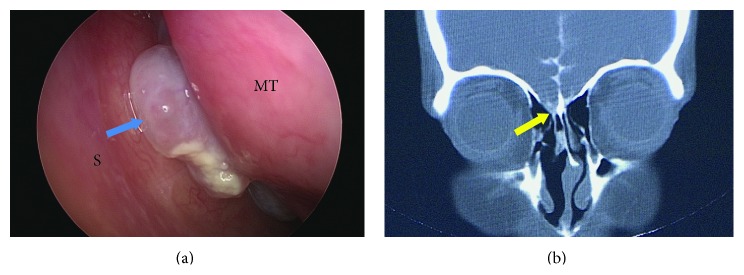
Patient number 2. (a) Left meningocele (arrow) between the nasal septum (S) and middle turbinate (MT). (b) CT myelography showing a defect at the level of the cribriform plate (arrow head).

All ten patients presented with cerebrospinal fluid leak (three cases on the left side and seven cases on the right), and three of them also had meningitis. The pathology results revealed seven meningoceles and three meningoencephaloceles. The requested imaging modality was chosen according to treating physicians' preferences. Six cases were investigated by CT and a defect at the cribriform plate was evident in all of them (Figure 2). A cephalocele was detected radiologically in four out of six CT scans and in the remaining two cases there was only a suspicion of cephalocele that was confirmed intraoperatively. CT myelography was used in three cases and MRI in one case; in these a cephalocele was evident radiologically (Table 1).

After reviewing the histological sections, seven cases out of ten were meningoceles and three cases showed evidence of neural tissue and were labeled as meningoencephaloceles as a final diagnosis.

As for the reconstruction of the base of skull defect, the temporalis fascia graft was used in all cases, in addition to fibrin glue (Tissucol) and Surgicel (Ethicon). In 2 cases where a larger defect was found, a thin bone harvested from the perpendicular plate of the ethmoid was applied and an inferior turbinate mucosa was laid over the bone. This has been effective in achieving a watertight seal in all cases. The follow-up period was between 6 and 38 months and no recurrence of symptoms was recorded in any of the patients. No adverse events or harms were encountered.

## 4. Discussion

The cause of encephaloceles remains unclear and several hypotheses have been considered [2]. The embryologic development of the skull helps in understanding the origin of encephaloceles. It could be due to failure of fusion at the level of cartilaginous neurocranium, the membranous neurocranium, or viscerocranium or it could be due to pneumatization of sinuses resulting in areas of weakness. Other possible causes are exposure to teratogens, environmental factors, genetic and familial causes, or other related intracranial hypertension to spontaneous CSF leaks [2, 7, 17].

The incidence is between 0.8 and 4 per 10,000 births and there is no sex predominance [1, 2]. Most cases of anterior meningoencephaloceles present in infancy and childhood and 50% of the basal subtypes are diagnosed in infancy [8]. Patients can present with different signs and symptoms such as nasal polyps, nasal obstruction, CSF rhinorrhea, seizures, and recurrent meningitis that is mainly because the defect in the skull will serve as route for pathogens from surrounding sinuses/air cells to reach the meninges [7–16]. Due to the rarity of the condition in adulthood, a high index of suspicion is mandatory. As such, CT scan is indicated to localize bony defect as the MRI will confirm the presence of herniated brain tissue [17, 29, 30]. In our case series all patients presented with CSF leak with or without meningitis and the cephalocele was detected radiologically in the preoperative settings in all 10 cases.

An increase in the intracranial pressure has been linked to spontaneous CSF leaks and cephaloceles. There appears to be an overlap in risk factors for idiopathic intracranial hypertension and spontaneous CSF leaks such as female gender and obesity. In fact, our data shows a large female to male ratio of 8 : 2 and Nyquist et al. have reported 32 patients with meningoencephaloceles of the anterior skull base area and found a statistically significant trend toward females (78.5%, *p* < 0.05).

Different methods to reduce intracranial pressure were discussed such lumbar drains, VP shunts, and acetazolamide [19]. The use of a lumbar drain to release any possible increased intracranial hypertension and therefore avoid a forcing pressure over the reconstructed material is a subject of controversy and many articles are published in the literature supporting or refuting the use of a lumbar drain in the case of meningoencephaloceles [27, 28]. Some support the idea of using a drain to avoid any spikes of increased intracranial hypertension [28]; others do not because it is not clear that it improves the closure rate and there is a fear of inducing pneumocephalus [18–27]. None of the CSF diversion techniques were used for our patients and no postoperative CSF leak or meningitis was noted.

As for the approach for surgery, craniotomy is the conventional treatment technique and several authors support it. It is argued that this technique provides a better visual field for the surgeon, allows the use of large flaps, and reduces risk of infections [5, 8]. However, previous studies have shown that it was successful in 70% of cases and was associated with higher morbidity, mortality, and recurrence rates [18]. Kubo et al. completed a series of 19 cases of transethmoidal meningoencephaloceles where 17 patients underwent the transcranial approach for excision and repair of the base of skull defect; 16 patients had a good outcome [8]. On the other hand, endonasal repair of skull base defects was introduced as a safe and efficient alternative with less postoperative morbidity. Schick et al. performed 136 endonasal duraplasties for frontobasal defects and reported a 94.9% success rate from the first surgical attempt [31]. Furthermore, in a prospective study, Nyquist et al. supported the endoscopic repair of nontraumatic anterior encephaloceles claiming a closure rate of 93.8% [19].

Patients with increased risk for recurrent CSF leak are those presenting with spontaneous CSF leaks [20]. In such condition, multilayer closure of the defect is of paramount importance to achieve a watertight seal and reduce the incidence of postoperative recurrence of CSF leak [21]. The different materials used for reconstruction include the fascia lata, fat, cadaveric pericardium, septal cartilage, turbinate mucosa, vascularized flaps, synthetic dural substitute (Dura-Gen, Integra LifeSciences Corp., Plainsboro, NJ), and Alloderm fascial graft [22]. Cellulose material (Surgicel, etc.), glue (fibrin glue, etc.), Clips (Nitinol clips), and balloon catheter can also be used in order to strengthen the reconstructed defect [23].

One of the major points of controversy is the technique of insertion of the graft. In general there are three main techniques of closure depending on where the graft is placed. The underlay technique consists of placing the graft between the dura and the bone at the edges of the defect. The overlay technique is placing the graft over both the dura and the bony borders of the defect. In a third method, two grafts are used for both overlay and underlay. No significant statistical difference is present when comparing the different techniques [21] but many authors favor the overlay procedure because it is technically less challenging and reduces the risk of olfactory fibers injury at the cribriform plate [24–27].

There are no comparative studies between one technique and another; almost all published data about the repair of a base of skull defect is based on personal experiences according to surgeon or center preferences. In our series, all 10 patients underwent endonasal repair of base of skull defect and the reconstructed material was laid as an overlay graft. The used surgical technique achieved favorable outcome, which further confirms the success of this technique and supports the findings of other series. None of our patients experienced a persistent leak and none had recurrence of meningitis. The endoscopic approach and reconstruction using a temporalis fascia graft have therefore achieved a 100% success rate with a total of 10 patients. Postoperative complications were limited to hyposmia, which progressively improved on follow-ups.

Comparative studies between one technique and another are needed to help better determine the superiority of any technique, if present, taking into consideration the cause of the defect, the size, the intracranial pressure, and surgeon's experience.

## 5. Conclusion

Spontaneous onset transethmoidal meningoencephalocele in the adult population remains a rare condition that requires a high index of suspicion for early and appropriate diagnosis. Multilayer reconstruction results in a high success rate and is advocated for treatment of such conditions.

## Figures and Tables

**Table 1:** 

Patient	Demographic (age, gender)	Presentation	Imaging	Diagnosis	Operation
1	42, female	Left CSFL	CT	Meningoencephalocele	TF, FG, PPE bone, and turbinate mucosa
2	43, female	Right CSFL	CT myelography	Meningocele	TF, FG, PPE bone, and turbinate mucosa
3	27, female	Right CSFL, meningitis	CT	Meningoencephalocele	TF, FG, and Surgicel
4	56, female	Right CSFL	MRI	Meningoencephalocele	TF, FG, and Surgicel
5	39, female	Right CSFL	CT myelography	Meningocele	TF, FG, and Surgicel
6	68, male	Right CSFL	CT	Meningocele	TF, FG, and Surgicel
7	43, male	Left CSFL	CT	Meningocele	TF, FG, and Surgicel
8	52, female	Right CSFL, meningitis	CT	Meningocele (x2)	TF, FG, and Surgicel
9	64, female	Right CSFL, meningitis	CT myelography	Meningocele	TF, FG, and Surgicel
10	56, female	Left CSFL	CT	Meningocele	TF, septal flap, and FG

TF: temporalis fascia, FG: fibrin glue, CSFL: cerebrospinal fluid leak, CT: computed tomography, and PPE: perpendicular plate of the ethmoid.
